# High Methionine Diet-Induced Alzheimer's Disease like Symptoms Are Accompanied by 5-Methylcytosine Elevated Levels in the Brain

**DOI:** 10.1155/2021/6683318

**Published:** 2021-04-07

**Authors:** Tingting Pi, Shenjiao Wei, Yongxuan Jiang, Jing-Shan Shi

**Affiliations:** Key Laboratory of Basic Pharmacology of Ministry of Education, Zunyi Medical University, Guizhou Province, China

## Abstract

**Background:**

Excessive or insufficient intake of methionine (Met) causes neuronal dysfunction, neurodegeneration, cerebrovascular dysfunction, vascular leakage, and short-term memory loss, which result in the occurrence of Alzheimer's disease- (AD-) like symptoms.

**Objective:**

To determine the relationship between high methionine diets (HMD) induced AD-like symptoms and 5-methylcytosine (5-mC) level.

**Methods:**

C57BL/6J mice were randomly divided into two groups: the control group (Maintain diets) and the model group (2% HMD). Mice were fed with 2% HMD for 9 weeks. Animals were weighed and food intake was recorded weekly. Open field test, nesting ability test, Y maze test, new object recognition test, and Morris water maze test were used to detect the motor, learning, and memory ability. Hematoxylin-eosin (HE) staining was used to observe the damage of cells in hippocampus and cortex. Immunofluorescence (IF) staining was used to detect the expression and distribution of amyloid-*β* 1-40 (A*β*_1-40_), amyloid-*β* 1-42 (A*β*_1-42_), and 5-methylcytosine (5-mC) in hippocampus and cortex. Western blotting (WB) was used to determine the expression of A*β* and DNA methyltransferases- (DNMTs-) related proteins in the cortex. Enzyme-linked immunosorbent assay (ELISA) was performed to detect homocysteine (Hcy) level (ELISA).

**Results:**

Feeding of HMD decreased the body weight and food intake of mice. Behavioral testing revealed that HMD caused learning, memory, and motor ability impairment in the mice. HE staining results showed that HMD feeding caused damage of hippocampal and cortical neurons, along with disordered cell arrangement, and loss of neurons. Furthermore, HMD increased the contents of A*β*_1-40_, A*β*_1-42_, and 5-mC in the hippocampus and cortex. WB results showed that HMD increased the expression of A*β* production-related proteins, such as amyloid precursor protein (APP) and beta-secretase 1 (BACE1), and decreased the expression of A*β* metabolism-related protein in the cortex, including insulin-degrading enzyme (IDE) and neprilysin (NEP). Additionally, the decreased expression of DNA methyltransferase1 (DNMT1) was observed in HMD-treated mice, but there was no significant change of DNMT3a level. ELISA results showed that HMD increased the levels of Hcy in serum.

**Conclusion:**

Our result suggested that the HMD can cause neurotoxicity, leading to AD-like symptoms in mice, which may be related to 5-mC elevated.

## 1. Introduction

With the development of nutritional epigenetics, it has been recognized that methionine (Met) metabolism plays an important role in epigenetic modification (DNA methylation, histone modification, chromatin remodeling, and noncoding RNA) [1]. Met is a sulfur-containing amino acid that cannot be synthesized by the body and must be obtained from an external source [2]. A high Met diet (HMD) affects brain functions and triggers neurotoxic effects, resulting in dementia-like neurodegeneration [3]. High Met, low folate, and low vitamin B6/B12 (HM-LF-LV) diets can cause short-term memory loss, cerebral vascular dysfunction, vascular leakage, neuronal impairment, and neurodegeneration [4]. Extensive evidence indicates that either excessive or insufficient intake of methionine can become harmful, further supporting the importance of a balanced diet based on a healthy lifestyle [5]. Nutrients can regulate metabolism and change disease susceptibility through DNA methylation pathways to maintain health and prevent disease occurrence [6]. Folic acid, choline, Met, and vitamin B (VitB) participate in one-carbon metabolism to promote S-adenosine methionine (SAM) production, enhancing DNA methylation and achieving the regulation of DNA methylation modification [7]. DNA methylation is one of the epigenetic regulations. Under the action of DNA methyltransferases (DNMTs), a methyl group covalently binds to the 5th carbon of cytosine residue of cytosine-phosphate-guanine (CPG) site to form 5-methylcytosine (5-mC) without the DNA sequence changed [8]. Studies have indicated that the increase of 5-mC is closely related to the occurrence of AD, which also revealed that *β*-amyloid (A*β*) metabolism and tau-related genes are regulated by DNA methylation [9].

However, the relationship among AD, methionine, and 5-mC has not yet been reported. Therefore, in the present study, we investigated that the symptoms of Alzheimer's disease (AD) induced by HMD may be modulated through regulating 5-mC.

## 2. Materials and Methods

### 2.1. Materials

Maintain diet and 2% of HMD were obtained from Synergetic Pharmaceutical Bioengineedring Co., Lt. (China). HMD was prepared by adding 2% Met to the Maintain diet. Amyloid precursor protein (APP, ab32136), beta-secretase 1 (BACE1, ab108394), insulin-degrading enzyme (IDE, ab109538), amyloid-*β* 1-42 (A*β*_1-42_, ab201061), DNA methyltransferase1 (DNMT1, ab188453), and DNA methyltransferase 3a (DNMT3a, ab188470) antibodies were purchased from Abcam (UK). Amyloid-*β* 1-40 (A*β*_1-40_, D8Q7I) and 5-mC (D3S2Z) antibodies were obtained from Cell Signaling Technology (USA). Neprilysin (NEP, D161012) antibody was obtained from Sangong Biotech (China). Glyceraldehyde-3-phosphate dehydrogenase (GAPDH, 10494-1-AP) and *β*-actin (66009-1-Ig) antibodies were obtained from Proteintech (China). HRP-conjugated affinipure goat anti-rabbit IgG (SA00001-2), HRP-conjugated affinipure goat anti-mouse IgG (SA00001-1), and CoraLite488- conjugated affinipure goat anti-rabbit IgG (SA00013-2) were obtained from Proteintech (China).

### 2.2. Animals

Male C57BL/6J mice (8-week-old, 20-25 g) were purchased from Changsha Tianqin Biotechnology. (Grade: specific pathogen-free [SPF], Certificate no.: SCXK (Xiang) 2014-0011SCXK). Mice were raised in SPF-grade animal facilities, with free access to food and water and a 12 h light/dark cycle (21–25°C, 8 : 00 am-8 : 00 pm). All animal procedures were approved by the animal experimental ethical committee of Zunyi Medical University.

### 2.3. Experimental Designs

After 2 weeks of adaptive feeding, mice were randomly divided into two groups (*n* = 8): control group (Maintain diet) and model group (2% HMD). The model group was given 2% HMD to achieve high levels of homocysteine (Hcy) in the plasma to cause disease status [10]. Food intake and body weight were recorded at a fixed time every week. After 9 weeks, behavioral tests were performed, and the mice were euthanized and then sacrificed.

### 2.4. Behavioural Test

#### 2.4.1. Open Field Test

The open-field experiment evaluates the motor ability of mice according to the free movement of mice [11]. Each mouse was put into the central zone of the open field tanks (40 cm × 40 cm × 40 cm), in which each mouse had 5 min of free movement, and a camera tracked the free movement of four mice in each reaction tank at the same time. The total distance of mice activity was recorded for statistical analysis.

#### 2.4.2. Nesting Ability Test

Nest building test is used to detect autonomous behavior and social ability. Each mouse was placed in its individual cages containing corn cob for 24 h to adapt to the environment. Then, one piece of cotton (5 cm × 5 cm × 1 cm) was placed in each cage as nesting material. The nest-building behavior was scored from 1 to 5 after 24 h according to previous study [12]: 1 = not noticeably touched; 2 = partially torn up; 3 = mostly shredded, but often no identifiable nest site; 4 = identifiable but flat nest; and 5 = perfect or nearly perfect nest. The scores were given by three independent observers blinded to treatment categories and pictures taken for documentation.

#### 2.4.3. Y Maze Test

Y maze is used to evaluate the spatial learning and memory ability of rodents by detecting the ability of spontaneous activity and autonomous selection [13]. The Y maze consists of three arms (40 cm × 8 cm × 15 cm) with an angle of 120° to each other. All mice were placed in one of the terminal identical arms to freely explore for 5 min without any added stress such as lights, sound, and food deprivation. In a series of explorations, the body completely entered the arm for standard entry. The movement speed and the total number of entering the closed arm (*n*) were recorded. The mice entering three different arms in succession were considered as the correct alternating reactions. Spontaneous alternation rate (%) = [correct number of alternation reactions/(*n* − 2)] × 100.

#### 2.4.4. New Object Recognition Test

The new object recognition experiment is a learning and memory test to evaluate visual recognition, which is based on the principle that animals tend to explore new objects [14]. Four reaction tanks (40 cm × 40 cm × 40 cm) of the same size were placed in a quiet room. Object A was a green cube, and object B was a brown cylinder. Adaptation period: mice were placed in the adaptation environment 5 min per day for 2 days to become familiar with the arena. Familiarization period: two identical objects A (A1 and A2) were placed in the same corner of four reaction tanks, and mice spent 5 min exploring the two identical objects freely (A1 and A2). Exploratory behavior was defined as directing the nose toward the object at a distance of less than 2 cm and/or touching the object with the nose. Test period: replaced A1 object with B and moved freely for 5 min. The time the mice spent exploring each object in the trials was recorded. The preferential index PI (PI = time spent exploring novel object/total exploration time) was used to evaluate the nonspatial recognition ability of mice.

#### 2.4.5. Morris Water Maze Test

Morris water maze is used to detect the ability of spatial learning and memory of mice [15]. A white circular pool (1200 cm in diameter) was filled with water (20-25°C and 25 cm deep), 1 cm taller than the hidden circular platform. The platform was located in one of the four equal quadrants with a computer system (equipped with a video camera) to automatically capture the data of each mice. During the experiment, mice were trained once a day for four consecutive days. Mice were individually placed into the water facing the wall and were allowed 60 s to find the hidden platform. If the mice stayed for more than 3 s after finding the platform within the 60 s, the experiment was terminated and the time for the mouse to find the platform was recorded, which was the escape latency (s). Otherwise, if the animal failed to find the hidden platform within 60 s, the mice were placed back onto the platform for the 20 s, and the escape latency was recorded as 60 s. The mean escape latency value of the three quadrants was used as the performance of the mice on a specific day. The platform was removed on the fifth day to conduct space exploration experiments. The first quadrant was selected as the water entry point. The number of times mice cross-platform position within the 60 s was recorded and analyzed. After the Morris water maze test, mice were euthanized, and blood samples from the retroorbital plexus were collected. One part of brain tissues was rapidly dissected to separate the prefrontal cortex and hippocampus on ice and stored at –80°C. Another part of brain tissues was then fixed in 4% paraformaldehyde solution and waxed.

### 2.5. Hematoxylin-Eosin (HE)

Briefly, brain sections (5.0 *μ*m thick) were dewaxed in xylene for twice (10 min each time) and rehydrated with graded alcohol (5 min each time). Sections were stained with hematoxylin solution for 20 min, followed by rinsing in distilled water for 1 min and differentiation of 1% hydrochloric acid ethanol for 3 s. Then, the sections were stained with eosin solution for 2 min followed by dehydration with graded alcohol. HE staining was performed to observe the histomorphology of the hippocampus and cortex. (Obtained a picture of the coronal section of the slice under a microscope and partition the hippocampus according to the brain atlas [16]. Under a 40x objective lens, collect images of hippocampal CA1, CA3, DG, and cortex areas. Conditions such as background and exposure time are set to be consistent.)

### 2.6. Immunofluorescence (IF) Staining

The brain sections (5.0 *μ*m) were in turn cleared in xylene, dewaxed with graded alcohol, and then the sections were washed two times with PBS (5 min each time). Sections were performed to antigen retrieval three times (6 min each time). Sections were dipped in 0.1% Triton-X-100 for 10 min and then blocked in goat serum for 30 min at 37°C. After washing again with PBS, sections were treated with the appropriate primary antibodies diluted in the blocking solution at 4°C overnight. The antibodies used were as follows: anti-amyloid-*β* 1-40 (A*β*_1-40_, 1: 500), anti-amyloid-*β* 1-42 (A*β*_1-42_, 1 : 200), and anti-5-methylcytosine (5-mC, 1 : 1000). The sections were then incubated with goat anti-rabbit IgG (H+L) 488 (1 : 500) for 30 min at 37°C, followed by washing twice with PBS. 4′,6-Diamidino-2-phenylindole dihydrochloride (DAPI) staining for 5 min and washing with PBS were subsequently performed. The slices were enclosed with antifluorescent. Images were acquired using an epifluorescence microscope (Olympus). The average fluorescence optical density of the hippocampus and cortex was analyzed by Image J. (The image acquisition method was the same as HE staining.)

### 2.7. Western Blot (WB) Analysis

Cortical tissues were collected and homogenized in radioimmunoprecipitation assay (RIPA) lysis buffer with freshly added proteinase inhibitors. After centrifugation (12000 rpm, 15 min), the protein concentration was determined with bicinchoninic acid (BCA) protein quantitative kit. Electrophoretic separation of 30 *μ*g of protein per hole in 8% or 10% sodium dodecyl sulfate polyacrylamide gel electrophoresis (SDS–PAGE) was conducted, followed by transferring onto polyvinylidene fluoride (PVDF) membrane. The membrane was blocked with 5% nonfat milk at room temperature for 4 h. The membranes were then incubated with primary antibodies (APP 1 : 5000, BACE1 1 : 7500, DNMT1 : 1000, DNMT3a 1 : 2000, NEP 1 : 1000, IDE 1 : 15000, GAPDH 1 : 50000, and *β*-actin 1 : 10000) at 4°C overnight. After washing with TBST, the membranes were incubated with HRP-conjugated affinipure goat anti-rabbit IgG (1 : 5000) or HRP-conjugated affinipure goat-anti mouse IgG (1 : 5000) for 1 h at room temperature. The visualization of blots was conducted in Gel Imaging using a chemiluminescence detection kit.

### 2.8. Enzyme-Linked Immunosorbent Assay (ELISA)

Hcy level in serum was detected by ELISA kit. Optical absorbance of each well was measured according to the manufacturer's instructions. A standard curve was used to calculate the Hcy level.

### 2.9. Statistical Analysis

All data were presented as mean ± SEM. Data were collected and analyzed by the GraphPad Prism 5 software. Statistical significance was tested by nonparametric *t*-test and two-way ANOVA. *p* < 0.05 was considered as statistically significant.

## 3. Result

### 3.1. Effect of HMD on Body Weight Gain and Food Intake of Mice

**Figure 1:**
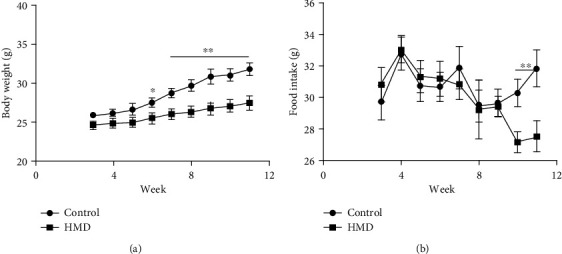
Effect of HMD on body weight and food intake. (a) The body weight of mice. (b) The food intake of mice. Note: the values were expressed as mean ± SEM (*n* = 8). ^∗^*p* < 0.05, ^∗∗^*p* < 0.01 vs. control group.

**Figure 2:**
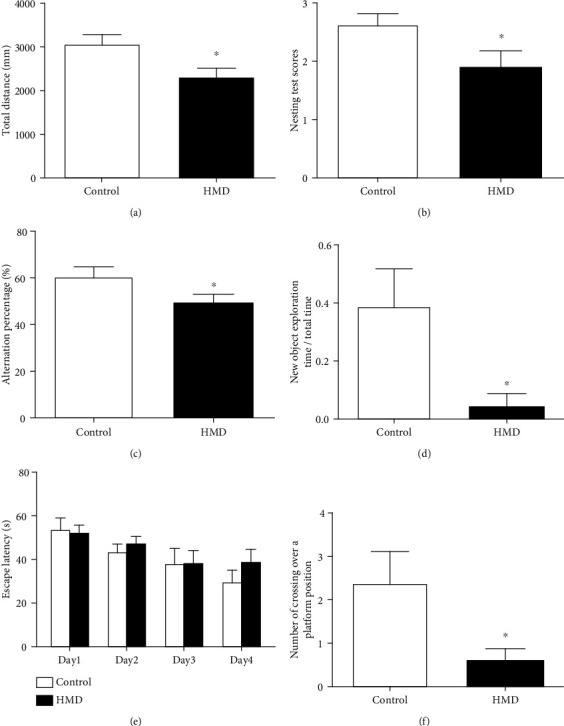
Effect of HMD on behavioral test of mice. (a) Total distance in the open-field test of each group. (b) Nesting test scores after 24 h of each group. (c) Alternation percentage % of each group. (d) New object exploration time/total time of each group. (e) The escape latency of each group. (f) Number of crossing over a platform position for 60 s. Note: the values were expressed as mean ± SEM (*n* = 8). ^∗^*p* < 0.05 vs. control group.

Feeding of HMD affected the body weight and food intake of mice. As shown in Figure 1, compared with the control group, the increase of body weight in the HMD group was significantly less in the 6th week (*p*  < 0.05, Figure 1(a)), which became more dramatic as the mice grew. The measurement of food intake showed that there was a significant decrease in the food intake of mice fed with HMD as compared to the control group (*p* < 0.05, Figure 2(b)). These results indicated that feeding with HMD decreased mice body weight gain and food intake.

### 3.2. HMD Impairs the Motor Function in the Open Field Test

**Figure 3:**
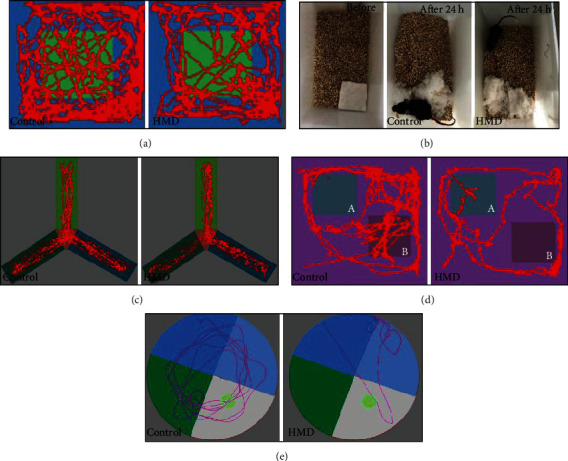
Representative photographs of behavioral test. (a) Representative tracing of open-field test in each group. (b) Representative photographs of nesting test in each group. (c) Representative tracing of Y maze test in each group. (d) Representative tracing of new object recognition test in each group. (e) Representative tracing of Morris water maze test in each group.

The open field test was designed to evaluate motor activity (Figure 3(a)). Through analyzing the total distance recorded in the open field test, we found that there were significant differences between the HMD and control groups (*p* < 0.05, Figure 2(a)), indicating that HMD-treated mice had a motor ability impairment as compared to the control group.

### 3.3. HMD Causes Learning and Memory Deficits

Nesting test scores were used to evaluate the effect of HMD on the nesting ability of mice in the present study. As shown in Figure 3(b), the nest-building ability was significantly different between the HMD group and the control group after 24 h (*p* < 0.05, Figure 2(b)). The results indicated that the feeding of mice with HMD resulted in the decrease in nesting ability.

The effect of HMD on spontaneous alternation behaviors was evaluated by the Y-maze test (Figure 3(c)). The HMD group showed a significant impairment in spontaneous alternation behavior when compared with the control group (*p* < 0.05, Figure 2(c)), indicating that HMD caused significant impairment of spontaneous alternation behavior.

The effect of HMD on visual recognition ability was evaluated by a novel object recognition test (Figure 2(d)). The PI of the HMD group was significantly decreased as compared with the control group when object A was replaced with the novel object B (*p* < 0.01, Figure 2(d)). This indicated that HMD caused significantly visual recognition impairment.

In the course of training period, the escape latency of mice in each group was getting shorter, and there was a difference on the 4th day, but it was not significant (*p* > 0.05, Figure 2(e)). In the space exploration test (Figure 3(e)), mice treated with HMD showed a significant reduction in the number of crossing over a platform position in the 60 s when compared with the control group (*p* < 0.05, Figure 2(f)). All these data demonstrated that the treatment of mice with HMD caused the learning and memory deficits of mice.

### 3.4. HMD Damages Neurons in the Cortex and Hippocampus Tissues

**Figure 4:**
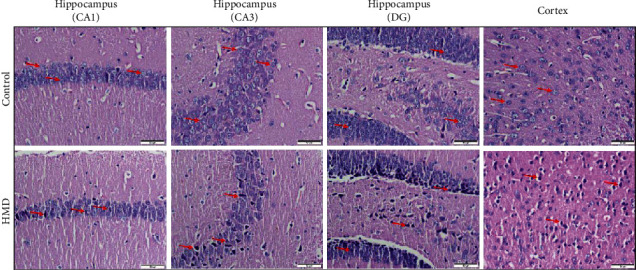
Images of HE staining. Effect of HMD on neuronal damage in the hippocampus and cortex. Sections of the hippocampus CA1, CA3, DG region, and cortex were obtained and stained with HE (magnification, 400x). (a) Showed representative hippocampal cytoarchitecture of CA1, CA3, DG, and cortex in each group.

HE staining was used to observe the neuronal damage of the hippocampus and cortex in the brains of mice. Compared with the control group, the neuronal cells were damaged in the HMD-treated group (Figure 4(a)). It was found that the staining of neurons was abnormal, pyramidal layer of archicortex structure was disordered, and nuclear shrinkage was present in the hippocampus and cortex of the HMD group mice (Figure 4(a)). These results showed that the feeding with HMD caused certain damage to the brain of mice.

### 3.5. HMD Increased A*β*_1-42_ Level in the Hippocampus and Cortex

**Figure 5:**
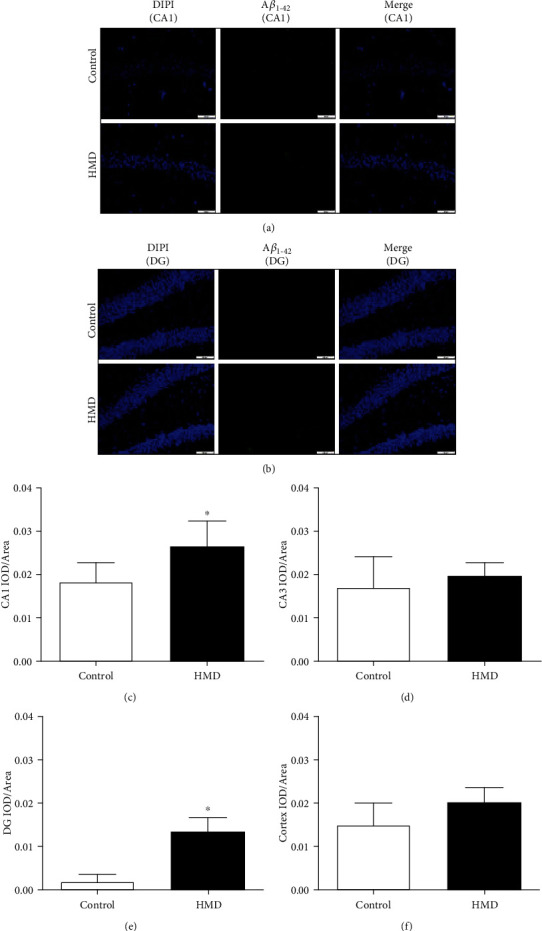
Effect of HMD on A*β*_1-42_ IF staining of hippocampal and cortex tissue in mice. Sections of the hippocampus CA1, CA3, DG region, and cortex were obtained and stained with IF (magnification, 400×). (a) Showed representative photomicrographs of A*β*_1-42_ IF staining results of each group in hippocampal CA1 region. (b) Showed representative photomicrographs of A*β*_1-42_ IF staining results of each group in hippocampal CA3 region. (c) Showed the CA1 IOD/area of each group. (d) Showed the CA3 IOD/area of each group. (e) Showed the DG IOD/area of each group. (f) Showed the cortex IOD/area of each group. Note: value was expressed as mean ± SEM (*n* = 4). ^∗^*p* < 0.05 vs. control group.

The A*β*_1-42_ content of HMD group in CA1 and DG region significantly increased when compared with the control group (*p* < 0.05, Figures 5(c) and 5(e)), while the CA3 and cortex region also has little difference (*p* > 0.05, Figures 5(d) and 5(f)). These results suggested that HMD could cause A*β*_1-42_ deposition in the hippocampus and cortex, resulting in brain damage.

### 3.6. HMD Increased A*β*_1-40_ Level in the Hippocampus and Cortex

**Figure 6:**
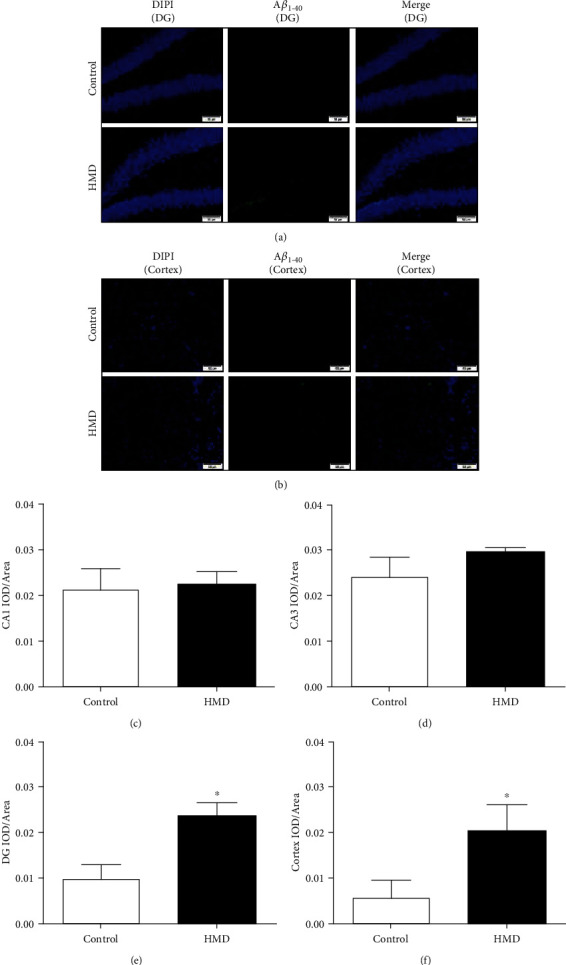
Effect of HMD on A*β*_1-40_ IF staining of hippocampal and cortex tissues in mice. Sections of the hippocampus CA1, CA3, DG region, and cortex were obtained and stained with IF (magnification, 400x). (a) Showed representative photomicrographs of A*β*_1-40_ IF staining results of each group in hippocampal DG region. (b) Showed representative photomicrographs of A*β*_1-42_ IF staining results of each group in the cortex. (c) Showed the CA1 IOD/area of each group. (d) Showed the CA3 IOD/area of each group. (e) Showed the DG IOD/area of each group. (f) Showed the cortex IOD/area of each group. Note: value was expressed as mean ± SEM (*n* = 4). ^∗^*p* < 0.05 vs. control group.

Compared with the control group, A*β*_1-40_ content in DG and cortex regions was significantly increased in the HMD group (*p* < 0.05, Figures 6(e) and 6(f)), and the content in the CA3 and CA1 regions also increased (*p* > 0.05, Figures 6(c) and 6(d)). These results indicated that HMD could cause A*β*_1-40_ deposition in the hippocampus and cortex, resulting in brain damage.

### 3.7. HMD Increased 5-mC Level in the Hippocampus and Cortex

**Figure 7:**
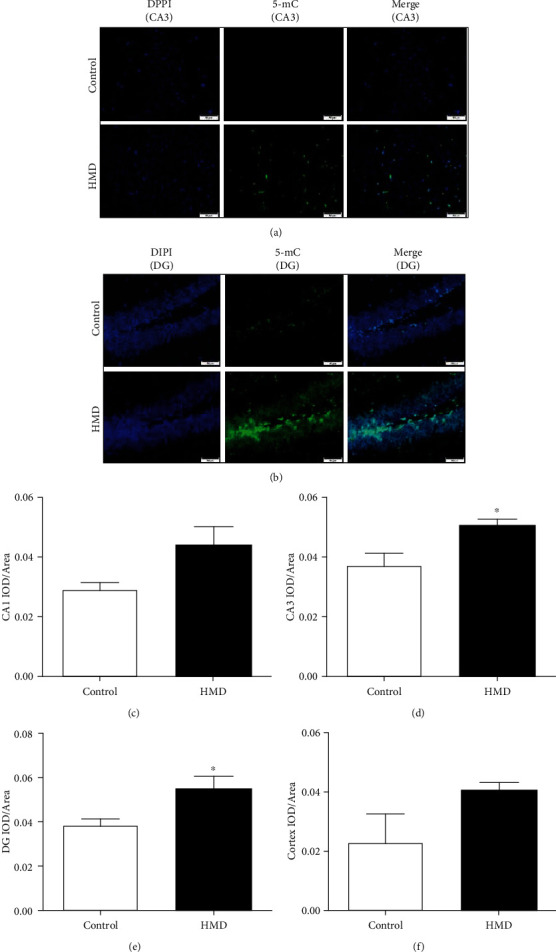
Effect of HMD on 5-mC IF staining of hippocampal and cortex tissues in mice. Sections of the hippocampus CA1, CA3, DG region, and cortex were obtained and stained with IF (magnification, 400x). (a) Showed representative photomicrographs of 5-mC IF staining results of each group in hippocampal CA3 region. (b) Showed representative photomicrographs of 5-mC IF staining results of each group in hippocampal DG region. (c) Showed the CA1 IOD/area of each group. (d) Showed the CA3 IOD/area of each group. (e) Showed the DG IOD/area of each group. (f) Showed the cortex IOD/area of each group. Note: value was expressed as mean ± SEM (*n* = 4). ^∗^*p* < 0.05 vs. control group.

The 5-mC content in the CA3 and DG regions was significantly increased in the HMD group when compared with the control group (*p* < 0.05, Figures 7(e) and 7(f)), and the content of 5-mC in CA1 and cortex areas also increased, but not significantly (*p* > 0.05, Figures 7(c) and 7(d)). These results showed that HMD caused an increase in the 5-mC level in the hippocampus and cortex.

### 3.8. HMD Upregulated A*β* Production-Related Proteins

**Figure 8:**
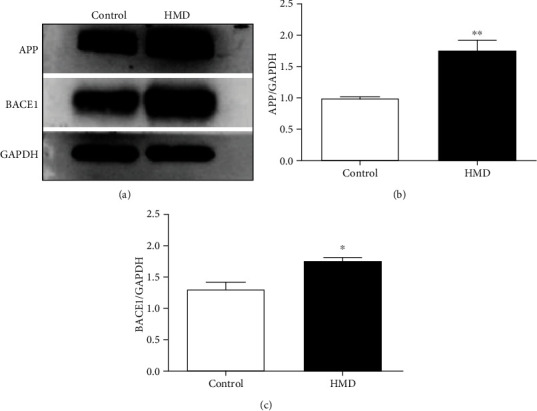
Effect of HMD on the expression of A*β* production-related proteins APP and BACE1 in cortex in mice. (a) Showed representative photomicrographs of APP and BACE1 proteins expression of each group in cortex. (b) The protein expression levels of APP in cortex. (c) The protein expression levels of BACE1 in cortex. Note: value was expressed as mean ± SEM (*n* = 4). ^∗^*p* < 0.05 vs. control group.

The expression of A*β* production-related proteins (APP and BACE1) in the cortex of the HMD group was significantly upregulated when compared with the control group (*p* < 0.05, Figures 8(b) and 8(c)). These data indicated that HMD could cause the overexpression of APP and BACE1 in the cortex.

### 3.9. HMD Downregulated A*β* Metabolism-Related Proteins

**Figure 9:**
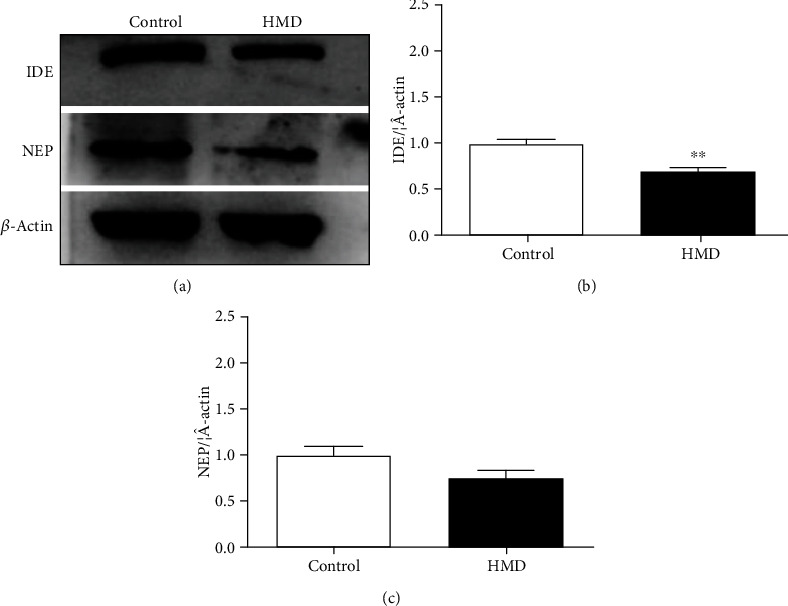
Effect of HMD on the expression of A*β* production-related proteins IDE and NEP in the cortex in mice. (a) Showed representative photomicrographs of IDE and NEP proteins expression of each group in cortex. (b) The protein expression levels of IDE in cortex. (c) The protein expression levels of NEP in cortex. Note: value was expressed as mean ± SEM (*n* = 4). ^∗^*p* < 0.05 vs. control group.

The expression of A*β* metabolism-related protein (IDE) in the cortex of the HMD group was significantly downregulated when compared with the control group (*p* < 0.05, Figure 9(b)). However, the protein NEP expression showed no significant decrease (*p* > 0.05, Figure 9(c)). These results showed that HMD could cause the IDE and NEP protein expression decline in the cortex.

### 3.10. Effect of HMD on the Expression of DNMT1 and DNMT3a

**Figure 10:**
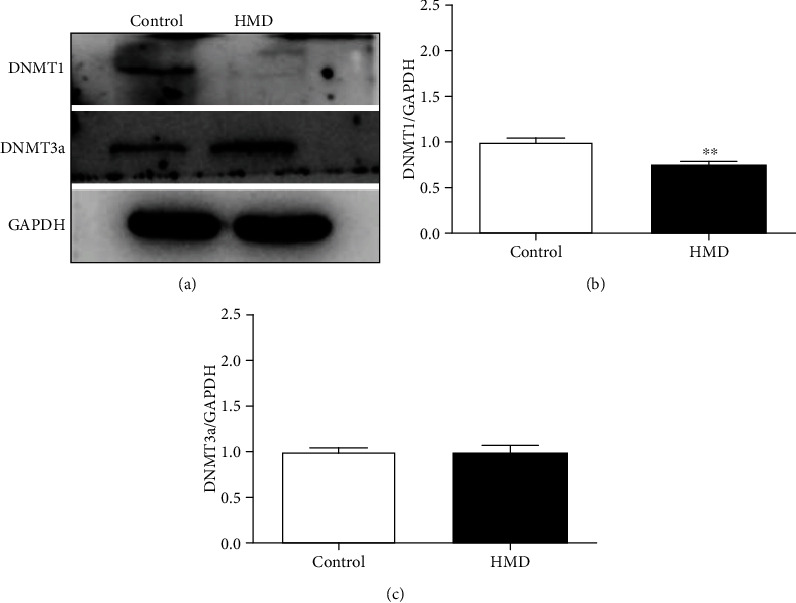
Effect of HMD on the expression of DNMT1 and DNMT3a proteins in cortex in mice. (a) Showed representative photomicrographs of DNMT1 and DNMT3a proteins expression of each group in cortex. (b) The protein expression levels of DNMT1 in cortex. (c) The protein expression levels of DNMT3a in cortex. Note: value was expressed as mean ± SEM (*n* = 4). ^∗^*p* < 0.05 vs. control group.

DNMT1 expression in the cortex of the HMD group was significantly downregulated when compared with the control group (*p* < 0.05, Figure 10(b)), while there was no significant change in the expression of DNMT3a (*p* > 0.05, Figure 8(c)). These results showed that feeding with HMD induced the overexpression of DNMT1 in the cortex.

### 3.11. HMD Increased Hcy Levels in Serum of Mice

**Figure 11:**
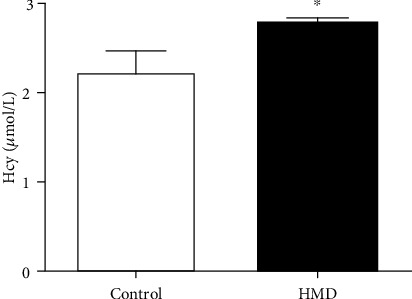
Effect of HMD on Hcy levels in serum of mice. (a) The Hcy levels in serum of mice. Note: value was expressed as mean ± SEM (*n* = 5). ^∗^*p* < 0.05 vs. control group.

As shown in Figure 11, compared with the control group, the Hcy level of the HMD group was significantly increased (*p* < 0.05), which indicated that HMD feeding resulted in an increase in Hcy level in mice.

## 4. Discussion

In the present study, we found that animals treated with an HMD exhibited an increase in the levels of A*β*_1-40_ and A*β*_1-42_ peptides as well as serum Hcy, all of which are hallmarks of AD. Alongside, these effects accompany the changes in the levels of 5-mC and cognitive deficits. Therefore, HMD intake might contribute to AD-like neurodegeneration that is related to 5-mC level.

Currently, a variety of animal models that develop AD-like symptoms have been employed in the research of AD, such as transgenic models, gene knockout models, and surgical models [17]. Although these models have been used to address some scientific questions, they possess some disadvantages including the lack of a unified operation, being invasive, expensive, and unstable, and having low modeling rates. At present, diet induction has been used to establish some animal models in the field of pharmaceutical research [18], which is convenient for breeding and economical in operation with animals being similar in their eating habits. Met is a methyl donor that cannot be synthesized in the human body and must be obtained externally in order to participate in the methylation reaction [19]. Studies have shown that excessive intake of Met can cause AD-like symptoms [3]. In the present study, we used HMD to induce AD-like symptoms to explore the relationship between 5-mC and AD, which provides certain advantages over other AD models. For instance, the genetic background has been changed in the transgenic mice models and gene knockout mice models. In the case of surgical modeling, operations may be inconsistent, which will cause different stimuli to individuals, leading to differences in methylation levels between different individuals in the same group. Because of the key advantage that the occurrence of methylation is based on intact gene sequences, diet-induced AD-like symptoms were used to study the changes in methylation levels. Moreover, the onset of AD usually occurs in one's late life, and its pathological development and progression [20] is a long-term gradual process. The feeding of HMD gradually changes the nutrient state of the body to induce AD-like symptoms, which is consistent with AD characteristics of slow and long duration of pathological development and severe illness.

Long-term HMD treatment inhibits growth and affects food intake, which is also accompanied by cognitive decline and neurotoxic effect [21]. However, how to confirm the length of the diet is very important for the study of AD-like symptoms caused by HMD. A short time diet cannot cause disease states and reduce the success rate of the model, while a long time diet will have an adverse effect on the body metabolism; it increases the success rate of the model but also causes a large accumulation of toxicity and increase the mortality rate [22]. Therefore, it is important to find an appropriate day of HMD. Excessive Met diet intake may impair gastrointestinal function to reduce food intake, which in turn lead to body weight loss. In the present study, we first determined the effect of HMD on body weight and food intake in mice. As the duration of HMD increased, these changes became more pronounced and the success rate of the model increased. Significant differences of body weight were observed at the 6th week (Figure 1(a)). Moreover, a difference of food intake was found at the 10th week (Figure 2(a)), which indicated that neurotoxicity may have occurred. Therefore, we hypothesized that AD-like syndrome was likely to occur at this time. The experimental results showed that HMD caused impairments in the nesting ability, spatial learning and memory, nonspatial learning and memory, and exercise ability, which proved that our hypothesis is correct, providing an important reference for similar experimental research in the future. Some mice consumed part of the food by grinding their teeth with food and thus caused unstable food intake, which is a limitation of the present study. Animal behavior plays an essential role in assessing animal models of cognitive dysfunction-related diseases and physiological mechanisms [23]. These function declines may be related to the neuronal damage and A*β* neurotoxicity in relevant brain regions (prefrontal cortex and hippocampus), which can be observed in HE staining and IF staining [24].

A*β* deposition is caused by excessive generation and reduced clearance of A*β*. Its toxic effects on neurons may be an important reason for impaired learning and memory, which are initiating factors and central links of AD [25]. A*β* is the core component of senile plaques and is a polypeptide composed of 39 to 43 amino acid residues. The presence of high levels of A*β* not only increases neuroinflammation and neuronal damage but also induces DNA damage [26]. A*β* fragments mainly exist in the form of A*β*_1-40_ and A*β*_1-42_ monomers. In cerebrospinal fluid, the concentration of A*β*_1-40_ is higher than A*β*_1-42_ [27], but A*β*_1-42_ is more toxic and easier to aggregate. A*β*_1-40_ and A*β*_1-42_ cause A*β* plaque deposition and senile plaques formation by folding from monomer to oligomer [28], causing a neurotoxic effect, synaptic damage, and neuronal death by forming fibers and gathering in the cerebral cortex, hippocampus tissue areas of the AD model [29]. A*β*_1-40_ and A*β*_1-42_ are the critical factors leading to the occurrence and development of AD [27]. After the feeding with the HMD, we found that the levels of A*β*_1-40_ and A*β*_1-42_ were significantly elevated in various brain areas of the HMD group, as evidenced in the HE staining and IF staining, indicating that an HMD caused damages in the hippocampus and cortex of mice, which was consistent with the results of other AD models studies [29].

APP metabolism includes the *α*-secretase pathway and *β*-secretase pathway, and the production of A*β* occurs in the *β*-secretase pathway. BACE1 is the key enzyme of the *β*-secretase pathway that plays an important role in the production of A*β* [30]. In AD patients, the expression level and enzyme activity of BACE1 are significantly increased, which is considered to be one of the body's natural defense lines against AD [31]. At the same time, A*β* clearance dysfunction will also result in its aggregation that can be degraded by various peptidases, with NEP and IDE being the most important ones. The expression level and activity of NEP and IDE in the AD brain are inhibited, which reduces A*β* degradation and aggravates the condition of AD [32]. In consistence with our results, the present study demonstrated that the expression of APP and BACE1 was significantly elevated in the HMD treated mice, while the expression of NEP and IDE proteins was downregulated, which suggested that the HMD caused AD-like symptoms were related to the production and metabolic pathways of A*β*.

Studies indicate that altered levels of 5-mC are associated with several diseases, including AD. Repeated measures and analyses of the data showed significant alterations in 5-mC in the early stages of AD, across multiple regions of the brain [33]. APP/PS1 mice showed decreased levels of 5-mC at 9 months of age throughout the hippocampus, while age-related increases in levels of 5-mC were found in wild-type mice [34]. On the other hand, there have been studies showing a significant negative correlation between 5-mC levels and amyloid plaque load in the hippocampus of AD patients [35]. Our experiment results showed that HMD increased A*β*_1-40_ and A*β*_1-42_ levels, which were also accompanied by the increases in serum Hcy levels and 5-mC levels in the hippocampus and cortex. The change in 5-mC level is different from previous studies, which may be related to the type of mice model and the diet. Our results showed that 5-mC is closely related to the AD-like symptoms induced by an HMD.

5-mC is the addition of a methyl group to the 5 carbon positions of cytosine with the help of DNMTs [36]. The activity of DNMTs is necessary for memory formation and storage [37]. DNMT1 is a maintained methyltransferase that is ubiquitously and highly expressed in proliferating cells and represents the main DNMT activity of somatic tissues during mammalian development [38]. It is reported that the elimination of DNMT1 in the mouse brain leads to insufficient methylation of cortical and hippocampal neurons, which leads to a decline in learning and memory in neurodegenerative processes [39]. In addition, DNMT3a is also closely related to learning and memory, which expression level of DNMT3a is significantly reduced in AD model mice. The hippocampal expression of DNMT3a decreases in aging mice. It has been demonstrated that an increase in hippocampal expression of DNMT3a can reverse memory deficits in aging mice, while knockout in young mice impairs memory formation [40]. Importantly, knockout of the DNMT1 and DNMT3a genes in mouse forebrain neurons will deteriorate animals' performance in the Morris water maze test [41]. Our results showed that the expression level of DNMT1 in the cerebral cortex of the model group mice significantly decreased, but the expression level of DNMT3a did not change. This indicates that the HMD may affect the learning and memory of mice by modulating the level of DNMT1 in the brain. DNMT3a has no significant changes because it is an active de novo methyltransferase, which is responsible for establishing DNA methylation patterns during early development and in germ cells [38]. Moreover, DNMT3a expression in different developmental stages and different tissues are different [42].

A long-term HMD will increase Hcy levels through one-carbon metabolism involving DNA methylation. High Hcy levels in the central nervous system are metabolic risk factors for neurodegenerative diseases and cognitive dysfunction, which cause learning and memory deficits by affecting hippocampal plasticity and synaptic transmission [43]. Hcy is an intermediate product of Met metabolism that plays a causal role in AD development [44]. HMD can increase the level of Hcy in the serum and eventually lead to hyperhomocysteinemia (HHcy) pathological condition where HHcy causes AD-like symptoms may be quite severe because the concentration of Hcy in the serum must be at least 15 *μ*mol/L to cause HHcy, while the concentration in the model group of this study was 2-3 *μ*mol/L which was much lower than the pathogenic concentration of HHcy [45, 46]. This indicates that before the occurrence of HHcy, the increase of Hcy concentration to a certain extent has led to the appearance of AD-like symptoms. In the present study, we determined the duration of HMD treatment according to the diet intake and body weight in order to prevent the appearance of HHcy to exclude other factors and that may confound the mechanism involved in HMD-induced AD-like symptoms. Moreover, dementia caused by HHcy is related to the neurotoxicity of amyloid, neurofibrillary tangles, and oxidative damage, which also may be related to abnormal methylation and disorder of gene expression [47]. HHcy could inhibit the proliferation of rat hippocampal neural stem cells and reduce the overall DNA methylation level by inhibiting the protein expression of DNMT1 and DNMT3a, which was consistent with the DNMT1 protein expression results of our experiment [48]. However, the expression of DNMT3a protein did not change, which may be related to the Hcy level in this experiment is lower than the level in the disease state of HHcy. Therefore, the relationship between disease states and DNA methylation at different Hcy levels is worthy of further study in the future.

In addition, this is the first study of chronic treatment that uses body weight and food intake to determine the number of weeks of the diet to focus on the neurobiological effects of HMD and its relationship with the occurrence of early neurodegenerative diseases. AD-like symptoms caused by HMD can also be observed in other studies. A diet rich in Met can cause the accumulation of A*β*_1-42_ peptides and increased tau phosphorylation by increasing Hcy level, which are all markers of AD. HMD promotes the accumulation of A*β* species, inflammation, oxidative stress, and cognitive deficits in wild-type mice [3]. Simultaneously, the increase in Hcy level caused by HMD has a similar stimulating effect on the structure and function of hippocampal, causing neurotoxicity and leading to AD-like symptoms [49]. Especially in the hippocampus, HMD will change the volume, tissue morphology, and metabolic profile of the hippocampus, which may eventually lead to neurodegenerative processes. It is generally believed that neurodegeneration is selective to different regions of the hippocampus, of which the CA1 region is the most sensitive. These processes are related to the morphological changes, the thinning of axons, and the decreased expression of neurons of the hippocampal CA1 area [50, 51]. In addition, the CA3 and DG areas of the hippocampus participate in spatial learning and memory, which are important for the maintenance of hippocampal function. Furthermore, it has been demonstrated that HMD reduces the neurons number in the CA3 and DG [52, 53], suggesting that these data help to understand the impact of HMD on changes in the nervous system. Although these effects are consistent with our results, they did not clarify the relationship between HMD-induced AD-like symptoms and 5-mC as in this work. We believe that HMD is potentially neurotoxic to different areas of the hippocampus and is related to 5-mC based on our result. Therefore, the results obtained in the present study provides us a new sight to elucidate the mechanistic linking between HMD induced AD-like symptoms and 5-mC.

In summary, we have found that HMD can cause AD-like symptoms, which confirmed that AD-like symptoms were associated with 5-mC level. The mechanism of HMD induce AD-like symptoms can explore in future studies, and these studies will be important in bringing about disease biomarkers and potential candidates for future therapeutics.

## 5. Conclusion

In the present study, we investigated the relationship between AD-like symptoms and 5-mC level in mice caused by an HMD. We conclude that HMD affects brain function in mice, resulting in AD-like symptoms, which are accompanied by the changes in 5-mC level that may play a role in the HMD induced AD-like symptoms.

## Data Availability

The datasets generated during and/or analyzed during the current study are available from the corresponding author on reasonable request.
